# The Protective Effects of Ciji-Hua’ai-Baosheng II Formula on Chemotherapy-Treated H_22_ Hepatocellular Carcinoma Mouse Model by Promoting Tumor Apoptosis

**DOI:** 10.3389/fphar.2018.01539

**Published:** 2019-01-08

**Authors:** Biqian Fu, Shengyan Xi, Yanhui Wang, Xiangyang Zhai, Yanan Wang, Yuewen Gong, Yangxinzi Xu, Jiaqi Yang, Yingkun Qiu, Jing Wang, Dawei Lu, Shuqiong Huang

**Affiliations:** ^1^Department of Traditional Chinese Medicine, Medical College, Xiamen University, Xiamen, China; ^2^Cancer Research Center, Xiamen University, Xiamen, China; ^3^Department of Traditional Chinese Medicine, Xiang’an Hospital of Xiamen University, Xiamen, China; ^4^College of Pharmacy, Rady Faculty of Health Sciences, University of Manitoba, Winnipeg, MB, Canada; ^5^Department of Physiology, Rady Faculty of Health Sciences, University of Manitoba, Winnipeg, MB, Canada; ^6^School of Pharmaceutical Sciences, Xiamen University, Xiamen, China

**Keywords:** Ciji-Hua’ai-Baosheng II Formula, chemotherapy, H_22_ hepatocellular carcinoma, Bcl-2, Bax, caspase-3, caspase-8, caspase-9

## Abstract

Ciji-Hua’ai-Baosheng II Formula (CHB-II-F) is a traditional Chinese medical formula that has been shown in clinical practice to relieve side effects of chemotherapy and improve quality of life for cancer patients. In order to understand the mechanism of its protective effects on chemotherapy, mice with transplanted H_22_ hepatocellular carcinoma were employed in this study. Ninety-two mice were injected subcutaneously with H_22_ HCC cell suspension into the right anterior armpit. After mice were treated with 5-fluorine pyrimidine (5-FU), they were divided into six groups as untreated group, 5-FU group, 5-FU plus Yangzheng Xiaoji Capsule group and three groups of 5-FU plus different concentrations of CHB-II-F. Twenty mice were euthanized after 7 days of treatment in untreated and medium concentration of CHB-II-F groups and all other mice were euthanized after 14 days of treatment. Herbal components/metabolites were analyzed by UPLC-MS. Tumors were evaluated by weight and volume, morphology of light and electron microscope, and cell cycle. Apoptosis were examined by apoptotic proteins expression by western blot. Four major components/metabolites were identified from serum of mice treated with CHB-II-F and they are β-Sitosterol, Salvianolic acid, isobavachalcone, and bakuchiol. Treatment of CHB-II-F significantly increased body weights of mice and decreased tumor volume compared to untreated group. Moreover, CHB-II-F treatment increased tumor cells in G_0_-G_1_ transition instead of in S phase. Furthermore, CHB-II-F treatment increased the expression of pro-apoptotic proteins and decreased the expression anti-apoptotic protein. Therefore, CHB-II-F could improve mice general condition and reduce tumor cell malignancy. Moreover, CHB-II-F regulates apoptosis of tumor cells, which could contribute its protective effect on chemotherapy.

## Introduction

Hepatocellular carcinoma is one of the most common cancers in the world and the third leading cause of cancer death. Moreover, it remains one of the most common malignant tumors in China. Surgery, radiotherapy and chemotherapy are among the common treatment options for patients with hepatocellular carcinoma. However, cancer recurrence, metastasis and side-effects of chemotherapy are the major concerns regarding to the prognosis of patients (Balistreri et al., 2005; Ciarimboli, 2012; Kudo et al., 2014; Tedore, 2015). Especially, side effects of chemotherapy such as fatigue, bone marrow suppression and gastrointestinal aversions lead to a dramatic decrease in quality of life (Ciarimboli, 2012; Xi et al., 2016; Ma et al., 2018). In recent years, traditional Chinese herbal medicine has been employed after chemotherapy to relieve adverse reactions and improve patient tolerance to chemotherapy (Liu, 2012). For instance, Shenling Baizhu powder is used to treat patients with leukemia receiving chemotherapy (Liu et al., 2012); Gujin Moji tablet has been shown to protect and promote bone marrow hematopoiesis in tumor-bearing mice with chemotherapy (Chen et al., 1998). Early studies revealed that first generation Ciji-Hua’ai-Baosheng Formula can prolong life of mice with transplanted ascitic H_22_ hepatocellular carcinoma, inhibit tumor growth, antagonize the decrease of white blood cells and platelets following chemotherapy, promote the production and activity of erythropoietin (EPO) and granulocyte-macrophage colony stimulating factor (GM-CSF), maintain the stability of peripheral blood cells, and improve immune functions (Xi et al., 2014; Cheng et al., 2016; Xi et al., 2018).

Professor Wang Yanhui, a prominent TCM practitioner, has used TCM to treat patients with malignant tumors. He believed that pathological changes in the internal environment of the body, such as productions of phlegm, dampness, and blood stasis initiate the occurrence of tumor. Clinically, malignant tumors are treated with surgery and/or a combination of radiotherapy and chemotherapy. Although tumor is targeted and removed, the internal environment of the body has not been changed fundamentally, and thus can induce tumor recurrence and metastasis (Lai et al., 2014). CHB-II-F is formulated based on Professor Wang’s extensive clinical experience (Wang and Xi, 2017), and is a second generation of formula refined from the original Ciji Hua’ai Baosheng Decoction (CHBD) (Cheng et al., 2016; Xi et al., 2018) without changing the principles of treatment in order to better facilitate its subsequent applications and further development. CHB-II-F is consisted of Radix Codonopsis, Semen Ziziphi Spinosae, Fructus Hordei Germinatus, Pericarpium Citri Reticulatae, Poria, Concha Ostreae, Bulbus Fritillariae Ussuriensis and Radix Salviae Miltiorrhizae. This formula is organized to help removing the pathological products while supplementing the body. Its aim is to restore balance to the internal environment, reduce tumor recurrence and metastasis, and improve the patient’s quality of life (Wang, 2004; Wang and Shen, 2004; Lai et al., 2014; Li et al., 2017; Liu et al., 2017).

5-fluorine pyrimidine, the most widely used pyrimidine drugs, has good curative effect on digestive tract cancers such as colon cancer, rectal cancer, gastric cancer, liver cancer, and other solid tumors, but has lots of side-effects that include hepatotoxicity and nephrotoxicity (Chinese Pharmacopoeia Commission [CPC], 2015b; Gelen et al., 2018). In China, Chinese physicians usually select 5-FU to combine with TCMs in order to reduce its bone marrow suppression, hepatorenal toxicity and gastrointestinal adverse reactions (Liu and Xie, 2018; Wang et al., 2018). YZXJC, a Chinese patent medicine approved by the State Drug Administration (SDA) of China for the treatment of primary hepatocellular carcinoma, is recorded in the Pharmacopeia of the People’s Republic of China (Chinese Pharmacopoeia Commission [CPC], 2015a), and it has been widely used in clinical practice in China and its efficacy has been clinically confirmed. Therefore, 5-FU and YZXJC were used as chemotherapeutic drug and herbal treatment drug in this study, respectively.

Apoptosis is a process of cell deaths featuring DNA fragmentation. Malignant tumor is related to not only increased cell proliferation, but also inhibition of cell apoptosis (Xi et al., 2016). Apoptosis is a complex cellular process regulated by many factors, such as Bcl-2 and Bax (Pan et al., 2014). From the current point of view, apoptosis is mainly regulated by two pathways: the pressure-induced pathway and the death receptor mediated pathway (Wang et al., 2011). The latter process depends on activation of death receptor, which triggers activation of caspases through formation of a death signal complex and promote apoptosis of tumor cells (Mcllwain et al., 2015).

Ciji-Hua’ai-Baosheng II Formula exhibits significant clinical benefits for patients after radiotherapy and chemotherapy. It is speculated that CHB-II-F may have anti-tumor effects, but molecular mechanism for its anti-tumor activity is not clear at present. The aim of this study is to investigate beneficial effects of the CHB-II-F formula on chemotherapy-treated mice, possible effects on apoptosis of tumor cells, and its possible anti-tumor mechanism.

## Materials and Methods

### Experimental Animals and Tumor Cells

Forty-six male and forty-six female special pathogen-free (SPF) Kunming mice, 18–22 g and aged 4–6 weeks, were purchased from Laboratory Animal Centre of Xiamen University in Xiamen, China (License No. SCXK (Min) 2017-005). The animals were given 1 week to adapt to the new environment before experimentation. All experimental procedures were approved by the Laboratory Animal Administration and Ethics Committee of Xiamen University (No. XMULAC 2012-0039). The H_22_ hepatoma cell suspension was provided by the Cancer Research Centre of Xiamen University (Xiamen, China).

### Experimental Drugs

**FIGURE 1:**
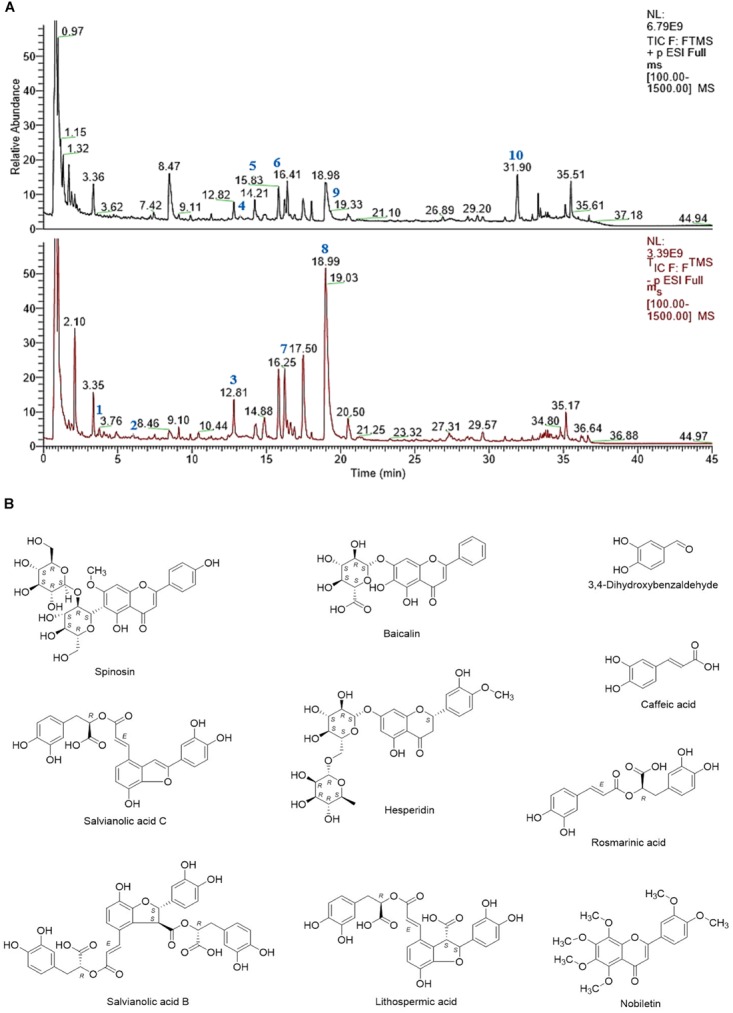
**(A)** UHPLC-MS chemical fingerprint of CHB-II-F. **(B)** Chemical structure of the major identified components of CHB-II-F. 3,4-Dihydroxybenzaldehyde (**1**), Caffeic acid (**2**), Spinosin (**3**), Baicalin (**4**), Salvianolic acid C (**5**), Hesperidin (**6**), Rosmarinic acid (**7**), Salvianolic acid B (**8**), Lithospermic acid (**9**) and Nobiletin (**10**).

Ciji-Hua’ai-Baosheng II Formula is composed of Radix Codonopsis, Semen Ziziphi Spinosae, Fructus Hordei Germinatus, Pericarpium Citri Reticulatae, Poria, Concha Ostreae, Bulbus Fritillariae Ussuriensis and Radix Salviae Miltiorrhizae (See Table 1) It was purchased from the Yanlaifu Pharmaceutical Co., Ltd. (Xiamen, China). Its chemical fingerprint (UHPLC) of CHB-II-F has been analyzed (See Figure 1) and the detailed methods of UHPLC is provided in the Supplementary Data. 5-fluorouracil, 5-FU (#1607261) was purchased from Tianjin Jinyao Pharmaceutical Co., Ltd. (Tianjin, China). 0.9% Sodium Chloride (#1702232) contained 100 mL in each ampoule. This product was produced by Fujian Tianquan Pharmaceutical Co., Ltd. (Longyan, China). YZXJC, with a product lot number of A1509006, was contained 0.39 g in each capsule. This product was produced by Shijiazhuang Yiling Pharmaceutical Co., Ltd. (Shijiazhuang, China).

**Table 1:** Herbal components of CHB-II-F.

Chinese name	Botanical name	Common name	Weight (g)	Voucher numbers	Part used
Dang Shen	*Codonpsis pilosula* (Franch.) Nannf., *Codonopsis pilosula* Nannf.var. *modesta* (Nannf.) L.T. Shen or *Codonopsis tangshen* Oliv.	Radix Codonopsis	10	160201	Root and rhizome
Fu Ling	*Poria cocos* (Schw.) Wolf	Poria	30	140130	Sclerotium
Mai Ya	*Hordeum vulgare* L.	Fructus Hordei Germinatus	20	131129	Germinated matured fruit
Chen Pi	*Citrus reticulata* Blanco	Pericarpium Citri Reticulatae	10	140213	Matured pericarp
Ping Bei Mu	*Fritillaria ussuriensis* Maxim	Bulbus Fritillariae ussuriensis	30	140130	Squamous bulb
Mu Li	*Ostrea gigas* Thunberg, *Ostrea talienwhanensis* Crosse or *Ostrea rivularies* Gould	Concha Ostreae	20	160201	Shell
Dan Shen	*Salvia miltiorrhiza* Bge	Radix Salviae Miltiorrhizae	50	161013	Root and rhizome
Suan Zao Ren	*Ziziphus jujuba* Mill. var. *spinosa* (Bunge) Hu ex H. F. Chou	Semen Ziziphi Spinosae	25	140130	Matured seed


### Main Reagents

In this study, we used the following materials: Annexin V-FITC/PI Apoptosis Detection Kit (product lot No. FXP018-100) and Cell Cycle Analysis Kit (product No. FXP021-100) were purchased from Beijing 4A Biotech Co., Ltd. (Beijing, China). Caspase-3 Antibody (product No. AB030), active caspase-3 antibody (product No. AC033), active caspase-8 antibody (product No. AC056), active caspase-9 antibody (product No. AC062), Bcl-2 antibody (product No. AB112), and Bax antibody (product No. AB026) were purchased from Beyotime Institute of Biotechnology (Jiangsu, China).

### Instruments

The following instruments were employed: YXJ-2 high speed refrigerated centrifuge (Xiang Yi Centrifuge Instrument Co., Ltd., Changsha, China), Rotary evaporator (Shanghai Yarong Biochemistry Instrument Factory, Shanghai, China), Freeze-dryer (Beijing Songyuan Huaxing Technology Development Co., Ltd., Beijing, China), ASP6025 Automated Vacuum Tissue Processor (Leica Co., Solms, Germany), Intellective Biological Microscope (Olympus optical Co., Ltd., Tokyo, Japan), Leica RM2016 histotome (Leica Co., Solms, Germany), Thermo UltiMate 3000 LC system and Thermo Q-Exactive system (Thermo Fisher Scientific, Bremen, Germany), Cosmosil CN column (Nacalai Tesque Co., Ltd., Kyoto, Japan), Beckman CytoFlex S Flow cytometry (Beckman Coulter, Kraemer Boulevard Brea, United States), and ONE-DScan program (Scanalytics inc., Fairfax, Va., United States).

### Medicinal Preparation

A total of 195 g of CHB-II-F crude herb was stored at -20°C until use. The herbs were soaked in 1950 mL water for 30 min and boiled, then decocted for 30 min to yield 200 mL. The solution was filtered through 8 layers of carbasus. The residue was soaked again in 1500 mL water and decocted for 30 min, and then filtered. The two filtered solutions were combined and concentrated with rotary evaporation (Shanghai Yarong Biochemistry Instrument Factory, Shanghai, China) at 58°C to 120 mL, then lyophilized with freezer-dryer (Beijing Songyuan Huaxing Technology Development Co., Ltd., Beijing, China). The lyophilized powder was sealed and stored at 4°C until use. The powder was reconstituted with distilled water to 3.25, 1.625, and 0.8125 g/mL for the CHB-II-F (H) [high dose of CHB-II-F], CHB-II-F (M) [medium dose of CHB-II-F], CHB-II-F (L) [low dose of CHB-II-F] treatment groups according to crude herb weight respectively. 5-FU was diluted by 0.9% sodium chloride to concentrations of 1 and 10 mg/mL. YZXJC was diluted by distilled water to 0.039 g/mL.

### Establishment of Chemotherapy-Treated H_22_ Hepatocellular Carcinoma Mouse Model

Hepatoma cells from Kunming mice with primary ascitic hepatoma cell H_22_ were collected under sterile condition and counted with a cell counter. Cells were then diluted with saline to a concentration of 2 × 10^7^ cells/mL and injected into SPF Kunming mice at a volume of 0.2 mL/10 g body weight subcutaneously at the right armpit. Seven days after injection, 92 mice have successfully developed tumors at the injected site. These mice then received peritoneal injections of 5-FU at 200 mg/kg to establish the chemotherapy-treated HCC model.

### Animal Groupings, Modeling and Drug Administration

Ninety-two chemotherapy-treated mice with H_22_ HCC were randomly divided into 6 study groups: physiologic saline (negative control), 5-FU (20 mg/kg), the YZXJC (0.78 g/kg) treatment, and three CHB-II-F [CHB-II-F (L), CHB-II-F (M), CHB-II-F (H), respectively] treatments. CHB-II-F groups received drug concentration of 0.8125, 1.625, and 3.25 g/mL [CHB-II-F (L), CHB-II-F (M), or CHB-II-F (H), respectively] by intragastric administration once a day for 14 days. The 5-FU group received peritoneal injection of 5-FU at 20 mg/kg (Li et al., 2003) at a volume of 0.2 mL/10 g once every other day for 14 days. The YZXJC group received intragastric injection at 0.039 g/mL once per day for 14 days. Each group had 12 mice. Another 20 mice were added to the physiologic saline group and CHB-II-F (M) group for component/metabolite analysis at mid-way of treatment.

### UHPLC-MS

On the 7th day of treatment, 10 mice from the saline and CHB-II-F (M) groups respectively were euthanized and peripheral blood was collected through retro-orbital bleeding. Serum was collected through centrifugation at 3000 rpm for 15 min and stored in -80°C until further analysis. Serum was washed with methanol at 1:4 ratio, mixed on shaker for 3 min and centrifuged for 15 min at 13000 rpm. The procedure continued until no pellet was observed after centrifugation, and the supernatant was then concentrated with blowing nitrogen. Blank serum of equal volume undergoing the same procedure was used as a control.

Serum from the CHB-II-F treated group were profiled by UHPLC coupled with a high-resolution electrospray ionization mass (HR-ESI-MS) detector. Prior to the MS detector, the UHPLC separation was performed over a Cosmosil CN column (2.6 μm, 250 mm × 4.6 mm i.d., 5 μm) on a Thermo UltiMate 3000 LC system. The mobile phase was acetonitrile (A) and water with 0.1% formic acid (*v/v*) (B), and the constituents were eluted by gradient according to the elution program as follows: A from 5 to 35% and B from 95 to 65% during 0–30 min, A from 35 to 100% and B from 65 to 0% during 30–35 min; A and B were kept at 100 and 0% repetitively during 35–45 min. The column was maintained at 35°C and eluted at a flow rate of 1 mL/min. The injection volume was 5 μL. A diode array detector (DAD) with detection wavelength of 254 nm and a high resolution ESI-MS detector were used to record the UHPLC chromatograms.

The MS spectra were recorded on a Thermo Q-Exactive system. The mass spectrometer of positive and negative ionizations was calibrated across *m/z* 100–1500 using the manufacture’s calibration standards mixture (caffeine, MRFA and Ultramark 1621 in an acetonitrile-methanol-water solution containing 1% acetic acid) allowing for mass accuracies of no more than 5ppm in the external calibration mode. The ionization voltage was 3.5 kV, and the capillary temperature was set at 300°C.

### Measurements of Tumor Volume, Weight, Inhibitory Ratio and Histology

The tumor length (mm) (a) and tumor width (mm) (b) were measured and recorded by a sliding calipers on the 7 and 14th day after treatment in H_22_ HCC mice. Tumor volumes were then calculated according to the formula: V (mm^3^) = 1/2 × a × b^2^. Mice were subsequently anesthetized by ethyl ether inhalation and sacrificed by cervical dislocation. Tumors were removed and weighed. The tumor inhibitory ratio (IR) was calculated as the average tumor weight of the untreated controls minus the average tumor weight of the treatment group/average tumor weight of untreated controls × 100%. A portion of the tumor tissue was fixed in a 10% neutralized formaldehyde solution for histological analysis. Paraffin sections with a thickness of 8 μm were developed and stained with H&E. Histologic changes were observed by light microscopy (100×) and recorded by photograph.

A portion of the tumor tissue (3–5 mm^3^) was fixed with 2.5% glutaraldehyde stationary solution and then 1% osmic acid for 2 h. After fixation, the tumor tissue was dehydrated with acetone gradiently, made into ultrathin sections, stained with uranium acetate and lead citrate, and observed by transmission electron microscope (6000×).

### Measurement of Tumor Cell Cycle by Flow Cytometer

Tumor tissue with a size of about 3 mm × 3 mm × 3 mm was resected, shredded, and then homogenized once on ice by Jiangyin-Jingying 5 ML glass grinder with cross-shaped handle (Jingying Glassware Co., Ltd., Jiangyin, China) with moderate force and speed for 3 s by hand. HBSS solution was added to the homogenate, filtered through a 400-mesh cell sieve, and the resultant cell suspension was collected. The cell suspension was centrifuged at 1000 rpm at 4°C for 5 min, and the pellet was collected. Approximately 2–5 × 10^6^ cells from the pellet was washed twice with 400 μL HBBS, centrifuged and re-suspended in 400 μL HBBS. Next, cell suspension was washed with 1 mL of 75% anhydrous ethanol drop by drop, and incubated overnight at 4°C in the dark. The next day, cells were centrifuged at 1000 rpm at 4°C for 5 min, and then resuspended in 400 μL HBSS. 20 μL of RNase A solution was added to the cell suspension and incubated at 37°C in a water bath for 30 min. Finally, cells were filtered by 400-mesh cell sieve, mixed with 400 μL of PI staining solution, incubated at 4°C for 60 min in the dark, and subjected to flow cytometry detection.

### Measurement of Tumor Cell Apoptosis by Annexin-V FITC/PI

A total of 1–5 × 10^5^ tumor cells were resuspended in 100 μL of 1× binding buffer. Annexin V-FITC (5 μL) and PI (5 μL) were added and incubated in the dark at room temperature for 10 min. Then, analysis of annexin V-FITC binding and PI staining were performed with a flow cytometer at excitation length of 488 nm.

### Caspase-3, Caspase-8, Caspase-9, Bcl-2 and Bax Protein Expressed in Tumor Tissues by Western Blot

A part of tumor tissues collected in RIPA buffer was used for protein analysis. Protein concentration was measured using bicinchoninic acid assay. Equal concentrations of proteins (100 μg) were separated by 8% SDS-polyacrylamide gel electrophoresis (SDS-PAGE) and transferred onto polyvinylidene difluoride membranes. The membranes were blocked with 1% casein solution for 2 h at room temperature. Primary antibodies against caspase-3, cleaved-caspase-3, caspase-8, cleaved-caspase-8, caspase-9, cleaved-caspase-9, Bcl-2 and Bax were incubated for 1 h at 37°C with the following dilution factors: caspase-3 (1:1000), cleaved-caspase-3 (1:1000), caspase-8 (1:500), cleaved-caspase-8 (1:500), caspase-9 (1:1000), cleaved-caspase-9 (1:1000), Bcl-2 (1:1000) and Bax (1:1000). Membranes were rinsed 5 times with PBST buffer for 3 min each before incubation with the peroxidase-conjugated streptavidin-secondary for 1 h at 1:10000 dilution. Protein levels were visualized with chemiluminescence solution and X-ray films. β-tubulin was used as a loading control. Films were scanned and the average optical densities of the bands were analyzed with ONE-DScan system.

### Statistical Analysis

Parametric data were expressed as means ± SD ($\bar{x}$ ± s). GraphPad Prism 5 software (GraphPad Software Inc., La Jolla, United States) was used to analyze the data. Statistical significance was determined by using one-way analysis of variance [One-way ANOVA]. Differences with *P* < 0.05 were considered significant.

## Results

### Analysis of CHB-II-F in Blood Serum

**FIGURE 2:**
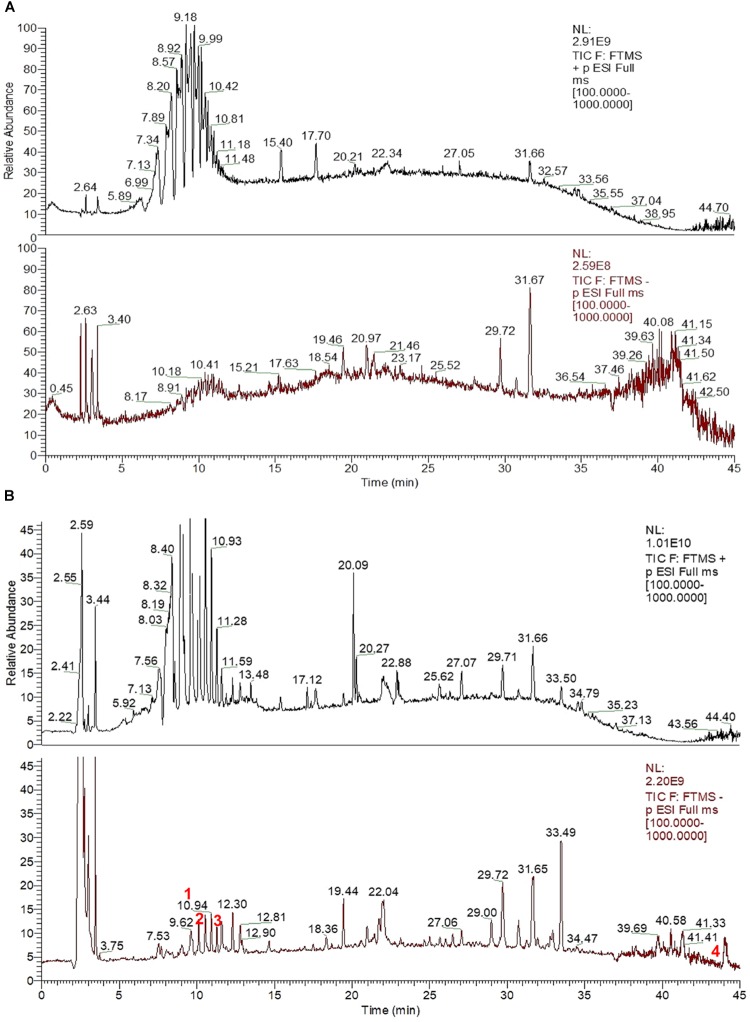
UHPLC-MS fingerprint chromatogram of CHB-II-F in blood serum [Blank serum ion map **(A)** and CHB-II-F-containing serum ion map of **(B)**].

**FIGURE 3:**
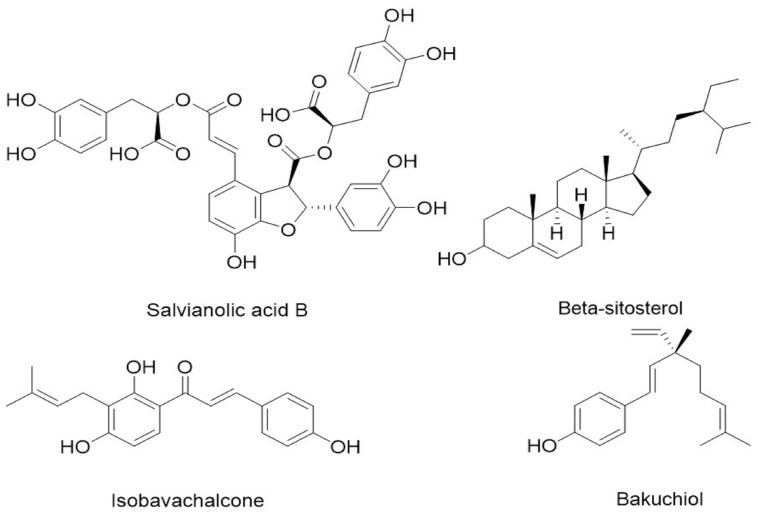
Chemical structure of the four identified components/metabolites of CHB-II-F dissolved in blood serum: Salvianolic acid B, β-Sitosterol, isobavachalcone and bakuchiol.

Ultra-high performance liquid chromatography -MS was used to analyze the serum of mice treated with CHB-II-F. Four components/metabolites (β-Sitosterol, Salvianolic acid B, isobavachalcone and bakuchiol) were identified by comparing the two serum ion profiles. The retention time at each phase was 9.62, 9.79, 11.27, and 43.95 min, respectively (Figures 2, 3).

### Effects of CHB-II-F on Body Weight Change, Tumor Weights and Volume

**FIGURE 4:**
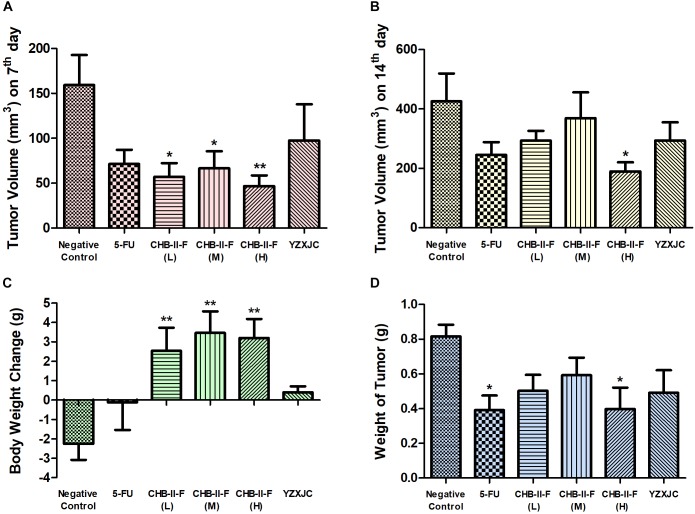
Effects of CHB-II-F on body weight, tumor weight and tumor volume of the chemotherapy-treated H_22_ hepatocellular carcinoma mouse model. **(A)** Tumor volume (mm^3^) on 7^th^ day; **(B)** Tumor volume (mm^3^) on 14^th^ day; **(C)** Body weight change (g); **(D)** Weight of tumor (g). Data were presented as the mean ± SD from 6 to 11 mice. Statistical analysis: ^∗^*P* < 0.05, ^∗∗^*P* < 0.01 compared with the saline group. No statistically significant differences were found when compared with the 5-FU group or between YZXJC and CHB-II-F groups (*P* > 0.05).

After 14 days of treatment, body weight in all CHB-II-F groups was significantly higher than that of untreated group (*P* < 0.01) (Figure 4C). Compared with the untreated group, tumor volume of CHB-II-F groups were significantly smaller on the 7th day and there was a trend of the higher dose the smaller body weight (*P* < 0.01) (Figure 4A). On the 14th day, both tumor weight and volume in the CHB-II-F (H) group were further decreased significantly (*P* < 0.05) (Figures 4B,D). The post-chemotherapy tumor inhibitory ratios (IRs) were 38.51, 27.51, 51.34, 52.08, and 39.85% in CHB-II-F (L), CHB-II-F (M), CHB-II-F (H), 5-FU and YZXJC groups, respectively.

### Effects of CHB-II-F on Pathology of Tumor Tissues

**FIGURE 5:**
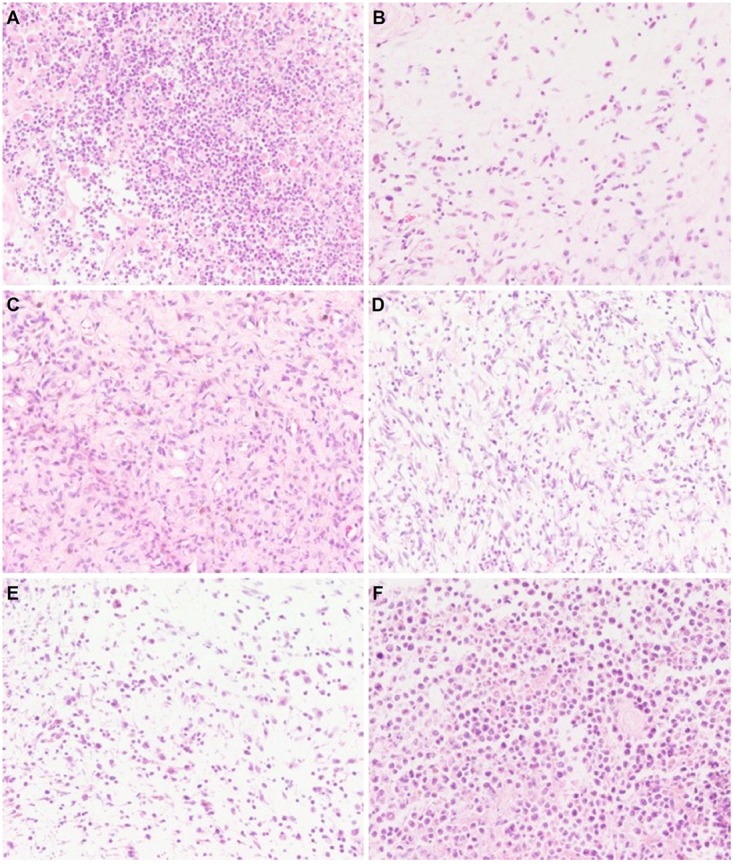
Effects of CHB-II-F on pathological changes of tumor tissue in the chemotherapy-treated H_22_ hepatocellular carcinoma mouse model. Sections were stained with hematoxylin and eosin (H&E) and viewed at a magnification of 400x. **(A)** Saline treated control (Negative control); **(B)** 5-FU treated control (20 mg/kg); **(C)** CHB-II-F (16.25 g/kg); **(D)** CHB-II-F (32.5 g/kg); **(E)** CHB-II-F (65 g/kg); **(F)** Yangzheng Xiaoji Capsule treated control (0.78 g/kg).

In the process of tumor removal, the tumor tissue was examined with the surrounding tissues. In the untreated group, the tumor was irregular in shape, large in volume and rough at the edges. After sectioning and staining with H&E, the nuclei were abnormally large, sizes of tumor cells varied, and the nuclei and cytoplasm were deeply stained. Tumor cells were arranged homogeneously and densely. In the 5-FU group, tumor cells were loosely arranged, cell number was decreased, and liquefactive necrosis and mitosis could be seen. Compared with both the saline and YZXJC groups, CHB-II-F treated groups showed scattered cell distribution, decreased tumor cell density, mitosis and heterogeneity within the tissue sections (Figure 5).

### Effects of CHB-II-F on Ultramicro-Pathology and Apoptosis in Tumor Cells

**FIGURE 6:**
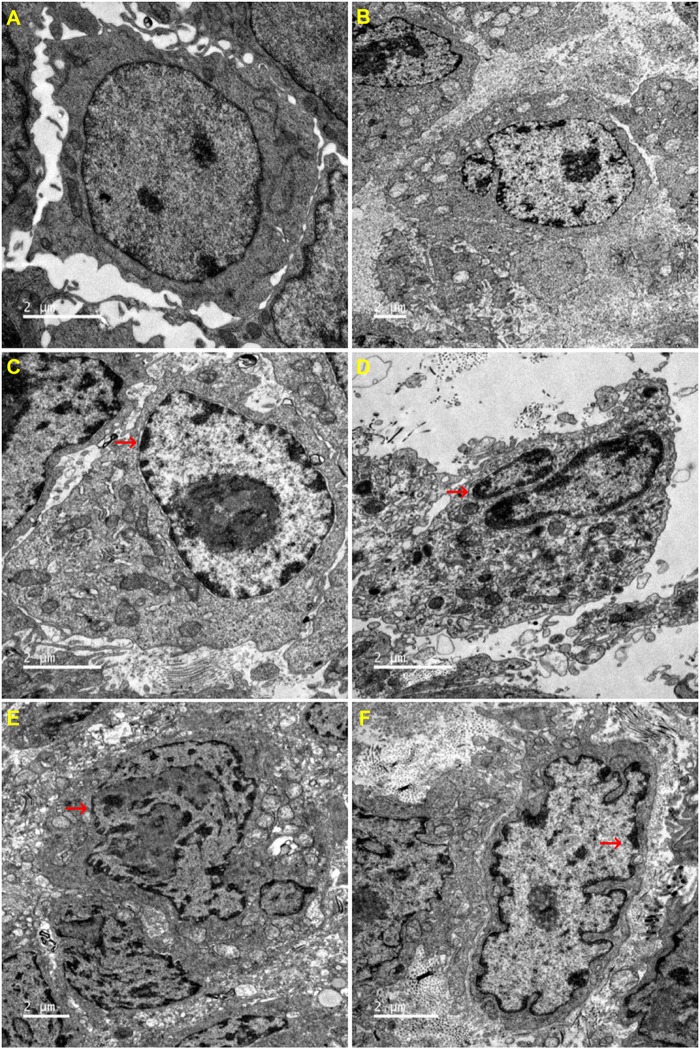
Effects of CHB-II-F on ultramicro-pathological changes and apoptosis in the chemotherapy-treated H_22_ hepatocellular carcinoma mouse model. Sections were stained with uranium acetate and lead citrate and viewed under 6000x magnification by transmission electron microscope. **(A)** Saline treated control (Negative control); **(B)** 5-FU treated control (20 mg/kg); **(C)** CHB-II-F (16.25 g/kg); **(D)** CHB-II-F (32.5 g/kg); **(E)** CHB-II-F (65 g/kg); **(F)** Yangzheng Xiaoji Capsule treated control (0.78 g/kg).

The ultrastructure of tumor cells in each group was observed by transmission electron microscope. Compared with the untreated group, all other groups exhibited different degrees of cell shrinkage and fragmentation, cytoplasmic condensation, irregular morphologies of nuclear and plasma membranes and condensed and peripheralized chromatin. Red arrows marked the locations of apoptotic bodies and pyknotic nucleus (Figure 6).

### Effects of CHB-II-F on Tumor Cell Cycle

**FIGURE 7:**
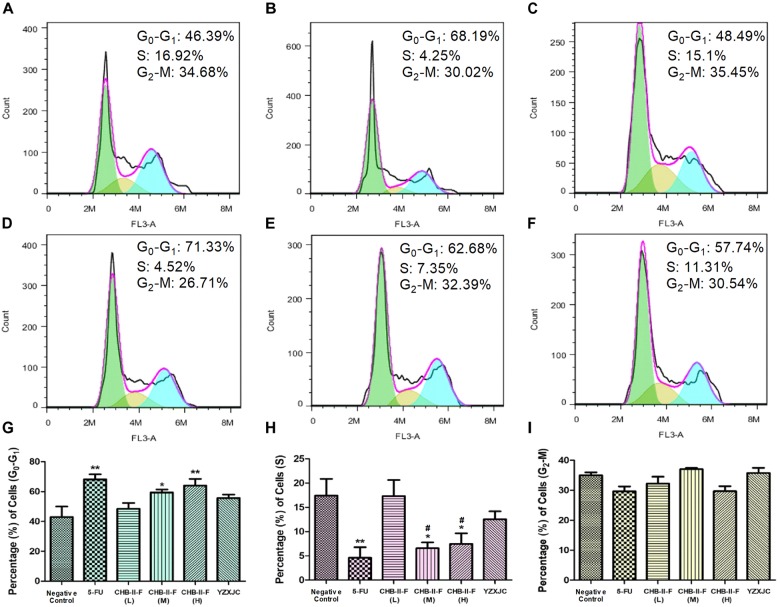
Effects of CHB-II-F on tumor cell cycle in the chemotherapy-treated H_22_ hepatocellular carcinoma mouse model. **(A)** Saline treated control (Negative control); **(B)** 5-FU treated control (20 mg/kg); **(C)** CHB-II-F (16.25 g/kg); **(D)** CHB-II-F (32.5 g/kg); **(E)** CHB-II-F (65 g/kg); **(F)** Yangzheng Xiaoji Capsule treated control (0.78 g/kg); **(G)** Gap phase 0-Gap phase 1; **(H)** Synthesis phase; **(I)** Gap phase 2-Mitotic phase. Data were presented as the mean ± SD from 6 mice. Statistical analysis: ^∗^*P*<0.05, ^∗∗^*P*<0.01 compared with the saline group; ^#^*P* < 0.05 compared with the CHB-II-F (16.25 g/kg) group. No statistically significant differences were found when compared with the 5-FU group or between YZXJC and CHB-II-F groups (*P* > 0.05).

The results of PI staining from flow cytometry revealed that the percentage of tumor cells in G_0_-G_1_ transition in the CHB-II-F (M) and CHB-II-F(H) groups was significantly higher, while the percentage in S phase was significantly decreased compared with the untreated group (*P* < 0.05; *P* < 0.01). However, the percentage of tumor cells in G_2_-M transition in all groups was not significantly different from that of the untreated group (Figure 7).

### Effects of CHB-II-F on Tumor Cell Apoptotic Index

**FIGURE 8:**
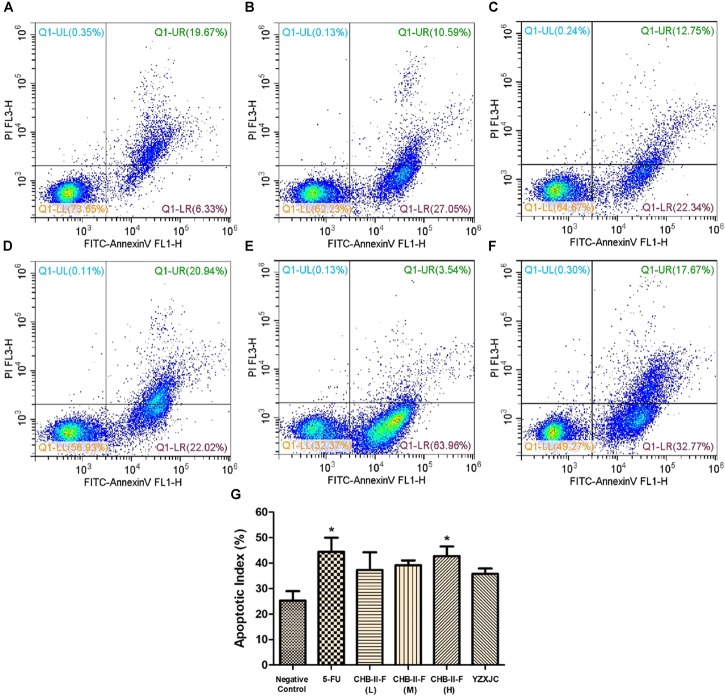
Effects of CHB-II-F on tumor cell apoptotic index in the chemotherapy-treated H_22_ hepatocellular carcinoma mouse model. **(A)** Saline treated control (Negative control); **(B)** 5-FU treated control (20 mg/kg); **(C)** CHB-II-F (16.25 g/kg); **(D)** CHB-II-F (32.5 g/kg); **(E)** CHB-II-F (65 g/kg); **(F)** Yangzheng Xiaoji Capsule treated control (0.78 g/kg); **(G)** Apoptotic index (%). Data were presented as the mean ± SD from 6 mice. Statistical analysis: ^∗^*P* < 0.05 compared with the saline group. No statistically significant differences were found when compared with the 5-FU group or between YZXJC and CHB-II-F groups (*P* > 0.05).

Annexin-V/PI staining was employed to detect early and late apoptotic cells. Figure 8 showed that the apoptotic index (%), defined as the percentage of apoptotic cells in all tumor cell population, were higher in all treated groups compared to the untreated group, with 5-FU and CHB-II-F (H) being statistically significant (*P* < 0.05).

### Effects of CHB-II-F on Expression of Caspase-3, Caspase-8, Caspase-9, Bax and Bcl-2 in Tumor Tissue

**FIGURE 9:**
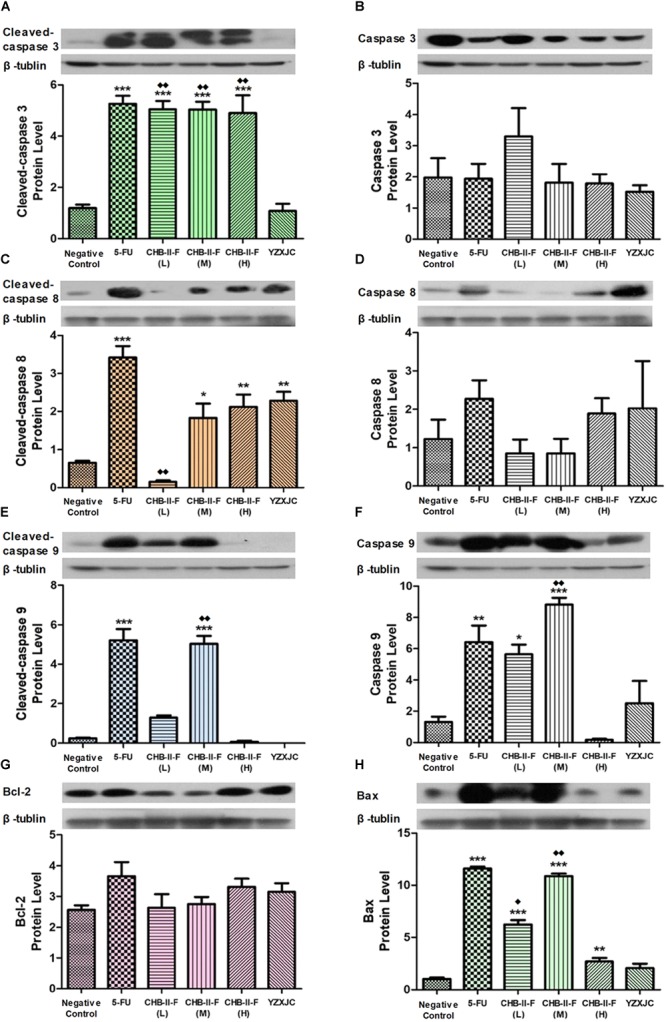
Effects of CHB-II-F on protein expression of caspase-3, caspase-8, caspase-9, Bax and Bcl-2 in tumor tissue of the chemotherapy-treated H_22_ hepatocellular carcinoma mouse model. **(A)** Cleaved-caspase 3 protein level; **(B)** Caspase 3 protein level; **(C)** Cleaved-caspase 8 protein level; **(D)** Caspase 8 protein level; **(E)** Cleaved-caspase 9 protein level; **(F)** Caspase 9 protein level; **(G)** Bcl-2 protein level; **(H)** Bax protein level. Data were presented as the mean ± SD from 6 mice. Statistical analysis: ^∗^*P* < 0.05, ^∗∗^*P* < 0.01, ^∗∗∗^*P* < 0.001 compared with the saline group; ^
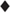
^*P* < 0.05, ^
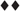
^*P* < 0.01 compared with the YZXJC group. (The same loading control was used for uncleaved and cleaved versions of the same protein, and for Bcl-2 and Bax for ease of comparison).

Western blotting showed that the expression of cleaved caspase-3 was significantly increased in 5-FU group as well as all the CHB-II-F-treated groups. The expression of cleaved caspase-8 was significantly increased in 5-FU and all treated groups except CHB-II-F(L) group. In addition, cleaved caspase-9 was increased in both 5-FU and CHB-II-F(M) groups. Compared to the untreated group, there was a slight decrease in the expression of apoptotic regulator, Bcl-2, in the CHB-II-F(L) and CHB-II-F(M) groups, but the expression of pro-apoptotic protein, Bax, was significantly higher in all three CHB-II-F groups. As a result, the ratio of Bax/Bcl-2 in all three CHB-II-F groups were higher (Figure 9).

## Discussion

Malignant tumor is the abnormal growth of cells in the human body, which has the ability to proliferate uncontrollably and metastasize. Millions of people are diagnosed with malignant tumors every year, and the incidence overall has been on the rise (Marusyk et al., 2012; Miller et al., 2016; Siegel et al., 2017; Chen et al., 2018). Malignant tumor in China alone accounts for about 21.8% of all cancer morbidity, among which, the incidence of hepatocellular carcinoma accounts for 50.5% and marks it as one of the major causes of cancer death in China and the third leading causes of cancer death in the world (Torre et al., 2015; Chen et al., 2017). Hepatocellular carcinoma is an aggressive disease with high malignancy, rate of recurrence and metastasis even after treatment, accompanied by short survival time and poor prognosis. At present, methods to control hepatocellular carcinoma include radiotherapy, chemotherapy, surgical resection, cryoablation, liver transplantation and interventional therapy, but patient outcomes have proven unsatisfactory (Ciarimboli, 2012; Xi et al., 2014; Wang et al., 2015). In China, the use of TCM as an adjuvant treatment for hepatocellular carcinoma has been shown to relief pain, reduce cytotoxicity of chemotherapy, improve symptoms of cancer, and control recurrence and metastasis (Song et al., 2016). Due to the relative low cost and ease of administration, TCM has gained recognition and used as an adjuvant therapy for chemotherapy (Chinese Society of Liver Cancer [CSLC], 2009). For instance, some Chinese medicine, such as the Yiqi Huayu Jiedu prescription, has been used as regular treatment for patients with hepatocellular carcinoma after radiotherapy and chemotherapy, which has been demonstrated to inhibit tumor angiogenesis by downregulating HIF-1α, Twist1, Bcl-2, MMP-2, MMP-9 and upregulating E-cad (Zeng et al., 2015). Radix Sophorae Flavescentis has been shown to inhibit the growth of lung adenocarcinoma cells *in vitro* and slow the growth of Lewis lung cancer cells and ascitic carcinoma cells of tumor-bearing mouse *in vivo* (Lin et al., 2009). Hydroxycamptothecin embolization combined with Shentao Ruangan pills can effectively improve the therapeutic effect of large hepatoma and improve patient survival (Lin et al., 2005). The aim of our experiment was to study the adjuvant effect of CHB-II-F on hepatocellular carcinoma after chemotherapy, and to explore the components/metabolites and mechanism of CHB-II-F in promoting cancer cell apoptosis.

Despite the aggressive and chaotic liver cancer phenotype, it often progresses in an orderly manner. Often, a cell acquires a mutation in its tumor suppressor gene or in other regulator of cell proliferation, causing it to enter an altered and malicious path (Hanahan and Weinberg, 2011; Shafie et al., 2013). Following that, changes in cell morphology, surface protein expression and cell cycle regulation quickly follow and ultimately lead to the development of tumor population and associated complications (Rieger, 2004). The ultimate goal of cancer treatment is to kill cancerous cells without affecting normal cells (Ma et al., 2015). With the rapid increase in scientific knowledge and technological advances, cancer mortality has been greatly reduced, but not without consequences (Holohan et al., 2013; Simard et al., 2013). While radiotherapy and chemotherapy kill rapidly dividing cells such as cancer cells, they inevitably cause damage to other normal growing cells in the body. It is therefore necessary to identify ways to target apoptosis to only cancer cells and spare normal cells (Shafie et al., 2013). Many TCMs have inhibitory effects on cancer cells, which mechanisms involve in promoting tumor cell apoptosis. Therefore, applying Chinese herbs to treat cancer is a wise choice (Newman and Cragg, 2012; Wang et al., 2015; Cao et al., 2017).

All eukaryotic cells have a defined cell cycle in which physiological processes such as growth, replication, division, senescence and death resume (King and Cidlowski, 1995). Cell cycle is controlled by a sophisticated and precise regulatory mechanism, and dysregulation leads to uncontrolled proliferation of cells and tumor formation (Wyllie et al., 1980). Whether a cell undergoes division or apoptosis is dictated by signals received during the cell cycle (Hartwell, 1992; Nurse, 1997). Usually, when a cell is blocked at a certain phase for prolonged period, apoptosis occurs. Many tumor chemotherapeutic agents treat tumor by inducing cell cycle-specific apoptosis (Collins et al., 1997). In this study, flow cytometry was used to analyze the effect of CHB-II-F on H_22_ hepatoma cells. We found that the percentage of tumor cells arrested in G_0_-G_1_ transition in the CHB-II-F (M) and CHB-II-F (H) groups were significantly higher. This shows that CHB-II-F may effectively influence cell proliferation and promote cell apoptosis. Although the IR of CHB-II-F (H) group was slightly inferior to the IR of 5-FU group, the body weight of mice in three CHB-II-F groups was increased, and higher than that in 5-FU group. The body weight loss of mice in 5-FU group was very obvious. These results indicated that after chemotherapy, combining with herbal medicine CHB-II-F could reduce 5-FU’s side-effects to a certain extent.

Cell apoptosis is very important for tumor control (King and Cidlowski, 1998). Apoptosis is the programmed death of cells intricately controlled by complex intracellular programs. There are two main pathways regulating apoptosis: the intrinsic stress-induced pathway and the death receptor-mediated pathway (Strasser et al., 2000). The former is induced by stress signals within the cell that promote the release of cytochrome C from the mitochondria, and the latter is activated by death receptor binding on the plasma membrane (Taylor et al., 2008). There are four major factors involved in the apoptotic pathway: Bcl-2 family of proteins, the caspases, heat shock proteins, and the p53 tumor suppressor gene. The Bcl-2 family are further divided into two groups: pro-apoptosis and anti-apoptosis. Bcl-2 itself is anti-apoptotic, and is mainly found in the mitochondria and cytoplasm. Bax, the pro-apoptotic counterpart, is mainly distributed in the cytoplasm and translocates to the mitochondrial membrane and disrupts mitochondrial integrity after receiving apoptotic signal. It is found that when a stress signal occurs, Bcl-2 and Bax form heterodimers, which decrease the availability of Bcl-2, and thus promote apoptosis of cells (Lee et al., 2014; Min et al., 2014). The caspases belong to the aspartyl specific cysteine protease family and play important roles in the signal transduction of apoptosis (Nuñez et al., 1998; Boyce et al., 2004; Xi et al., 2016). Based on the signaling cascade, caspases can be divided into two categories: primers and effectors, among which caspase-8 and caspase-9 belong to the primer group, while caspase-3 belongs to the effector group (Riedl and Shi, 2004; Fulda, 2009; Tait and Green, 2010; Reubold and Eschenburg, 2012; Würstle et al., 2012). Based on signals from the surrounding environment, a cell activates the apoptotic pathway by first cleaving and activating the cytoplasmic caspase-8, which then activates caspase-3. Pathways downstream of caspase-3 function to suppress inhibitors of apoptosis and inhibit the activity of proteases associated with DNA repair and mRNA splicing, which ultimately lead to chromatin condensation, nuclear and DNA fragmentation, cytoplasmic membrane blebbing (Fulda and Debatin, 2006). Mitochondria are the major regulatory organelles of apoptosis induced by stress signal (Wu et al., 2014). The opening of permeability transition pores in the inner and outer mitochondrial membranes leads to the release of cytochrome C, apoptosis activating factor-1 and other caspase activators. Cytochrome C and apoptosis activating factor-1 activate caspase-9, which in term activates caspase-3 and a variety of endonucleases to induce morphological changes of apoptosis (Fulda and Debatin, 2004; Gu et al., 2014). Activated caspase-3 is the key to inactivation of other apoptotic proteins, which cause alterations in cell structure, cell cycle and DNA repair (Bratton and Salvesen, 2010). Uncontrolled cell proliferation and apoptosis are reasons for the occurrence of tumor, thus regaining control of these pathways are the fundamental methods to manager tumor growth (Shao et al., 2015). In this study, the expression of Bcl-2, Bax, caspase-3, caspase-8 and caspase-9 in tumor tissues were detected by western blotting, and the cell cycle makers and apoptotic index of tumor cells were detected by flow cytometry. The results showed that protein expression of Bcl-2 in mice treated with CHB-II-F had a downward trend, while expression of Bax were increased. The expressions of activated caspase-3, -8 and -9 in tumor tissue were also upregulated, thus promoting apoptosis of tumor cells. These results demonstrated that the appropriate concentration of CHB-II-F can inhibit expression of anti-apoptotic factors and block proliferation of tumor cells, while at the same time increase the pro-apoptotic factors to induce tumor cell death. Thus, the efficacy of chemotherapeutic drugs in the treatment of hepatocellular carcinoma was enhanced.

Based on the UPLC results, four components/metabolites from CHB-II-F were found in the serum of treated mice: β-Sitosterol, Salvianolic acid, isobavachalcone and bakuchiol. It is worth noting that two of the monomers have anti-tumor effects. β-Sitosterol is one of the sterols components widely found in various vegetable oils, nuts and plant seed. It has many important physiological and pharmacological functions, such as lowering blood lipids, anti-inflammatory and cytotoxic effects (Zhang et al., 2011; Xiao et al., 2015; Yuan et al., 2015). It has been suggested that β-Sitosterol can effectively inhibit the growth of hepatoma carcinoma cells, decrease mitochondrial membrane potential, induce apoptosis and the up-regulation of Bcl-2, cleaved caspase (3, 8, 9) expressions (Zhang et al., 2011). β-Sitosterol can also induce apoptosis of human leukemia cell line U937 by activating caspase-3 protease and regulating Bax/Bcl-2 ratio (Park et al., 2007). Salvianolic acid B is a water-soluble compound found in the plant Salvia miltiorrhiza, which is its most active ingredient (Durairajan et al., 2008; Cao et al., 2012). Salvianolic acid B has been reported to have anti-tumor properties against cancers of the lung, liver, breast and prostate and squamous cell carcinoma of the head and neck. The proposed mechanisms include inhibition of nuclear gene transcription, inhibition of tumor angiogenesis and induction of tumor cells apoptosis with low toxicity to normal cells (Zhao et al., 2007; Hao et al., 2009; Zhao et al., 2010; Zhang and Lu, 2010). Isobavachalcone is a flavonoid with anticancer, anti-inflammatory, antibacterial and other biological activities (Kuete and Sandjo, 2012; Wang et al., 2012). Apoptosis of HepG2 cells can be induced by isobavachalcone, which was found to be related to an upregulated expression of Bcl-2 family proteins. Alternatively, it can also induce apoptosis of neuroblastoma by the intrinsic mitochondrial pathway (Nishimura et al., 2007; Wang et al., 2012). Bakuchiol is a monoterpenoid phenolic component with many pharmacological activities, such as antibacterial, antineoplastic, and anti-inflammatory actions (Jiang et al., 2010; Huang et al., 2014). Studies have shown that bakuchiol can inhibit breast cancer cells through inducing apoptosis and S phase arrest (Li et al., 2016). These components/metabolites of CHB-II-F found in the serum have known inhibitory effects on tumor, which provided the basis for exploring CHB-II-F effect on apoptosis of tumor cells. CHB-II-F may be down-regulating the expression of Bcl-2 and up-regulating the expression of Bax, while activating both caspase-3 and caspase-9, fixing tumor cells in G_0_-G_1_ transition, increasing the rate of apoptosis of rumor cells and stimulating cells to initiate the process of apoptosis. Morphological hallmarks of apoptosis were observed in the CHB-II-F groups, thus further confirmed the anti-tumor effect of the medicine.

In addition to its anti-tumor effects, CHB-II-F has been applied in clinics for many years, which has demonstrated repeatedly to reduce the side effects of gastrointestinal tract and improve dietary intake. Therefore, future studies can focus on identifying the underlying mechanisms. In addition, clinical trials in human patients treated with CHB-II-F would provide further confirmation on the beneficial effects observed in the current study.

## Conclusion

Four major components/metabolites of CHB-II-F have been identified in serum of mice treated with CHB-II-F. CHB-II-F treatment increased food intake, reduced pathological changes in tumor tissues and inhibited proliferation of tumor cells in the chemotherapy-treated HCC mouse model. The mechanistic action of CHB-II-F may be related to regulating expression of apoptotic factors and promote apoptosis of tumor cells.

## Author Contributions

SX and YanhW participated in the study design. BF and SX wrote the manuscript. YG, YX, and JY critically revised the manuscript. BF, XZ, YanaW, YQ, JW, SH, and DL carried out the experiments. BF and SX also performed the statistical analysis.

## Conflict of Interest Statement

The authors declare that the research was conducted in the absence of any commercial or financial relationships that could be construed as a potential conflict of interest.

## Abbreviations

5-FU: 5-fluorine pyrimidine
CHB-II-F: Ciji-Hua’ai-Baosheng II Formula
H&E: hematoxylin and eosin
HCC: hepatocellular carcinoma cells
TCM: traditional Chinese medicine
UHPLC: ultra-high performance liquid chromatography
YZXJC: Yangzheng Xiaoji capsule
