# Supplementary material for: The Protective Effects of Ciji-Hua’ai-Baosheng II Formula on Chemotherapy-Treated H22 Hepatocellular Carcinoma Mouse Model by Promoting Tumor Apoptosis
Source: Front Pharmacol. 2019 Jan 8;9:1539. doi: 10.3389/fphar.2018.01539 (PMC6331466; doi:10.3389/fphar.2018.01539)
Supplement: Supplementary file 1 [file Data_Sheet_1.docx]

*UHPLC-MS*

The chemical constituents of CHB-II-F extraction were profiled by ultra-high performance liquid chromatography (UHPLC) coupled with a high resolution electrospray ionization mass (HR-ESI-MS) detector. 10 mg lyophilized powder was dissolved in 1 mL of ultrapure water through ultrasonic method. The solution was filtered with 0.22 µm nylon filter membrane before injection into the UHPLC. The UHPLC separation was performed over a C18Kinetex column (100×2.1 mm i.d., 2.6 µm, Phenomenex Inc., Torrance, USA) on the Thermo UltiMate 3000 LC system (Thermo Fisher Scientific, Bremen, Germany). The mobile phases were acetonitrile (A) and 0.1% formic acid with water (*v/v*) (B). Samples were eluted by gradients according to the elution program as follows: A from 5% to 35% and B from 95% to 65% during 0-30 min, A from 35% to 100% and B from 65% to 0% during 30-35 min, A and B were kept at 100% and 0% respectively during 35 to 45 min. The column was maintained at 35 ℃ and eluted at a flow rate of 0.3 mL/min. The injected volume was 5 µL. A diode array detector with detection wavelength of 254 nm, and a high resolution ESI-MS detector were used to record the HPLC chromatograms. After UHPLC, samples were analyzed by MS spectra on a Thermo Q-Exactive system. The mass spectrometer with positive and negative ionizations was calibrated across *m/z* 100-1500 using the manufacturer’s calibration standards mixture (caffeine, MRFA and Ultramark 1621 in anacetonitrile-methanol-water solution containing 1% acetic acid) allowing mass fluctuation of no more than 5 ppm in the external calibration mode. The ionization voltage was 3.5 kV, and the capillary temperature was set at 300 ℃.
